# In Vivo Cross-sectional Characterization of Cerebral Alterations Induced by Intracerebroventricular Administration of Streptozotocin

**DOI:** 10.1371/journal.pone.0046196

**Published:** 2012-09-25

**Authors:** Audrey Kraska, Mathieu D. Santin, Olène Dorieux, Nelly Joseph-Mathurin, Emmanuel Bourrin, Fanny Petit, Caroline Jan, Marion Chaigneau, Philippe Hantraye, Pierre Lestage, Marc Dhenain

**Affiliations:** 1 Centre National de la Recherche Scientifique, URA 2210, Fontenay-aux-Roses, France; 2 Commissariat à l'Energie Atomique et aux Energies Alternatives, Direction des Sciences du Vivant, Institut d'imagerie biomédicale, Molecular Imaging Research Center, Fontenay-aux-Roses, France; 3 Institut de Recherches Servier, Croissy-sur-Seine, France; INSERM U894, France

## Abstract

Cerebral aging is often associated with the occurrence of neurodegenerative diseases leading to dementia. Animal models are critical to elucidate mechanisms associated to dementia and to evaluate neuroprotective drugs. Rats that received intracerebroventricular injection of streptozotocin (icv-STZ) have been reported as a model of dementia. In these animals, this drug induces oxidative stress and brain glucose metabolism impairments associated to insulin signal transduction failure. These mechanisms are reported to be involved in the pathogenesis of Alzheimer's disease and other dementia. Icv-STZ rats also display memory impairments. However, little is known about the precise location of the lesions induced by STZ administration. In this context, the present study characterized the cerebral lesions induced by two-doses of icv-STZ by using high-field magnetic resonance imaging to easily and longitudinally detect cerebral abnormalities and by using immunohistochemistry to evaluate neuronal loss and neuroinflammation (astrocytosis and microgliosis). We showed that, at high doses, icv-STZ induces severe and acute neurodegenerative lesions in the septum and corpus callosum. The lesions are associated with an inflammation process. They are less severe and more progressive at low doses. The relevance of high and low doses of icv-STZ to mimic dementia and evaluate new drugs is discussed in the final part of this article.

## Introduction

Cerebral aging is associated with the occurrence of neurodegenerative diseases leading to dementia such as Alzheimer's disease (AD). These diseases represent critical public health issues and intensive research is required to provide new treatments that counterbalance mechanisms leading to dementia. Animal models are critical to evaluate such therapies and one of these models relies on the intracerebroventricular administration of streptozotocin (icv-STZ). The rationale for this model is two-fold. First, strong evidences suggest alterations of cerebral insulin signaling [1] and a reduced brain glucose metabolism due to insulin signal transduction failure in AD [2], [3], [4]. STZ is a diabetogenic substance widely used in diabetes research to induce insulin depletion after intra-peritoneal injection [5]. After icv administration, STZ alters enzymes involved in the glycolytic cerebral metabolism of glucose [6], [7], [8] and insulin/IGF pathway therefore leading to an insulin resistance brain state that mimics some of the cerebral alterations occurring in AD [9], [10], [11], [12]. Second, icv-STZ administration also induces oxidative stress [12], [13], [14], [15], another characteristic of AD and other dementias [16], [17], [18], [19]. Icv administration of STZ has functional consequences; indeed several studies have shown behavioral alterations in icv-STZ animals. For example, icv-STZ treated animals display spatial [11], [20], working, episodic [21], and reference memory alterations [7]. Currently, the icv-STZ model is widely used to evaluate neuroprotective properties of various compounds such as anti-inflammatory drugs (cyclooxygenase inhibitors [22], Jun N-terminal kinase inhibitors [23], antioxidants (curcumin [24], S-allyl cysteine [25], selenium [26]), acetylcholinesterase inhibitors (donepezil, tacrine [21], [27], [28]), antidiabetic drugs (pioglitazone, [29]), cholesterol-lowering drugs (statins [30], [31] and others like adrenergic alpha and beta receptors antagonists (carvedilol [32]), protease inhibitors [33] or type-1 phosphodiesterase inhibitors (vinpocetine [13]).

Despite this wide litterature on the use of icv-STZ models, many protocols of icv administration of STZ have been proposed. Indeed, some studies are based on a single administration of STZ at either 1 mg/kg [34], [35] or 3 mg/kg [15], [29]. Intermediate doses from 1 to 3 mg/kg [11], [25] and/or multiple administration [28], [36] as well as very high doses (40 mg/kg [10]) have also been investigated. Also, the delays used to evaluate the injected animals vary from one week [10], [21] to six months [11] after icv-STZ administration. This raises questions concerning the effects of icv-STZ on cerebral pathology: Do different doses of STZ induce similar lesions or not? Are those lesions widespread or focalized on specific brain structures? How do these lesions evolve with time? The optimal use of icv-STZ models requires a better understanding of these questions.

Here we used high-field magnetic resonance imaging to follow-up in a cross-sectional way the cerebral lesions induced by icv-STZ injections at two different doses (1 and 3 mg/kg). We showed that STZ leads to a dose-dependant cerebral atrophy process preferentially affecting the septal area as well as the corpus callosum. Immunohistochemistry revealed gliosis associated to the described lesions and its severity increased with dose. Because of the dose dependant-effect that we reported, we suggest that low and high doses of STZ should be used in different applications.

## Materials and Methods

### Animals

Animal experiment procedures were performed in strict accordance with the recommendations of the EEC (86/609/EEC) and the French national committee (decree 87/848) for the care and use of laboratory animals. The research was conducted under the authorization number 91–326 from the “Direction Départementale des Services Vétérinaires de l'Essonne” and the Internal Review Board of the URA CEA CNRS 2210. The study was performed on male Wistar rats weighting 250–300 g (Charles River laboratory, France).

### Treatments and icv administrations

Rats were injected bilaterally through intracerebroventricular (icv) stereotactic injection (2 µL in each ventricle) with either a streptozotocin solution ([2-deoxy-2-(3-(methyl-3-nitrosoureido)-D-glucopyranose)], STZ, Sigma-Aldrich USA, dissolved in citrate buffer) at 1 or 3 mg/kg (treated group, n = 6) or a citrate buffer solution 0.05 M pH4.5 (control group, n = 6) one time. Citrate buffer is essential to correctly dissolve STZ (see Results). The solutions were freshly prepared just before the injection to avoid decomposition of the drug. Briefly, animals were anaesthetized with a mixture of ketamine (15 mg/kg) (Imalgene1000®, Merial) and xylazine (1.5 mg/kg) (Rompun® 2%, Bayer) through intraperitoneal administration. Rats were then placed into a stereotactic apparatus and were injected icv either with the solvent or the STZ solution (stereotactic coordinates relative to bregma, dura mater and inter-hemispheric scissure : antero-posterior: -0.7 mm; ventral: −3.6 mm, lateral: ±1.5 mm [37]).

### Longitudinal follow-up of cerebral atrophy by MRI

#### In vivo MR imaging

Brain images were recorded on a 7T Varian spectrometer by using a four channels surface coil (RapidBiomed) actively decoupled from the transmitting birdcage probe (RapidBiomed). Briefly, animals were anaesthetized by isoflurane (4% for induction and 1–2% for maintenance). Respiration rate was monitored to insure animal stability until the end of the experiment. Body temperatures of the rats were maintained by using an air-heating system. Two-dimensional fast spin-echo images were recorded with an isotropic nominal resolution of 230 µm (TR/TE/effective TE = 10000/14.3/14.3 ms, RARE-factor = 4). MR images were zerofilled to reach an apparent in-plane resolution of 115 µm.

#### Morphometric analyses

Morphometric analyses were based on the evaluation of intracranial, septal and striatal volumes as well as thickness of the cortical layer at the level of the parietal cortex and thickness of the white matter at the level of the corpus callosum. We chose these regions based on two criteria. First, they are close to the lateral ventricles where STZ was injected. Second, they were easy to outline because of their clear limits, leading to high reliability in the measurements.

**Figure 1:**
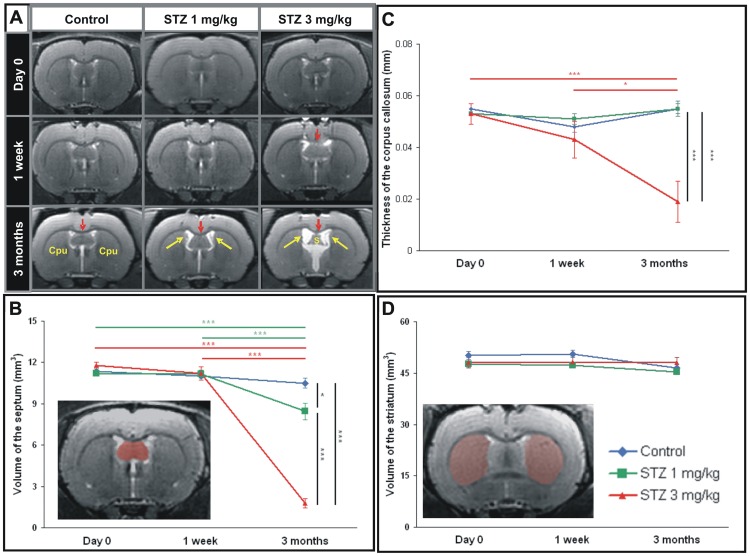
MRI evaluation of the effect of streptozotocin at 1 and 3 mg/kg in rats one week and three months following icv injection. A. A dilation of the ventricle (white signal on MRI, yellow arrows), an atrophy of white matter (red arrows) and septal alterations are visible after icv-streptozotocin. They are obvious in the 3 mg/kg treated animals. B. Quantification of the septal volumes showing an atrophy process in both treated groups. C. Thickness of the corpus callosum in controls, 1 mg/kg and 3 mg/kg treated-animals before the streptozotocin administration, one week and three months post-injection. D. Quantification of the striatal volumes showing no atrophy whatever the groups. Examples of the regions of interest used to outline the septum and striatum are showed in B and D, respectively. Values are mean±SEM. *: p<0.05; **: p<0.01; ***: p<0.005. S: Septum. Cpu: Striatum.

Volume analyses were performed using Anatomist® software (http://brainvisa.info/index_f.html). Before morphometric analysis, the brain images from the different rats were rotated to be positioned in a similar orientation. Each structure was manually outlined on coronal sections thanks to a digitizing tablet (Anatomist drawing tools) based on carefully evaluated criteria. The quality of the delineation of each structure was checked by examination of axial sections. The criteria to outline each structure were as follows. 1) The septal region was outlined from nine coronal slices where the boundaries were easily identified. The most caudal of the nine slices taken into account was the first slice showing the interventricular foramen (reference to bregma: anteroposterior -0.90 mm [37]).The lateral borders consisted of the two lateral ventricles, and the dorsal border was the corpus callosum. The ventral limit was defined by a virtual line between the two tips of the ventricles (Fig. 1B). 2) The striatum was outlined from nine coronal slices. The most caudal slice was the slice where the commissura anterior crosses the sagittal plane (reference to bregma: anteroposterior −0.30 mm [37]). The lateral borders consisted of the lateral ventricles and the limit of the striatum, and the dorsal border was the corpus callosum (Fig. 1D). 3) The intracranial volume, which included the brain and surrounding cerebrospinal fluid (CSF) were outlined from the most rostral part of the frontal pole to the most caudal part of the occipital pole. The high contrast between intracranial structures and other tissues made the delineation easy. An average of 70 coronal sections was outlined for each animal. Intracranial volume allowed correction for possible differences in structure volumes resulting from differences in total brain size. More precisely, for each structure of a given animal (A), the reported volume is equal to the measure of the volume of the structure divided by the intracranial volume for the rat (A), multiplied by the average intracranial volume from all of the rats.

The thicknesses of the parietal cortical layer and corpus callosum (white matter) were also evaluated on MR images. These analyses were performed using ImageJ software (http://imagej.nih.gov/ij/; National Institutes on Health NIH, USA) on the coronal section where the commissura anterior crosses the sagittal plane (reference to bregma: anteroposterior −0.30 mm, [37]). Thickness of the parietal cortex was defined at the level of the left and right primary somatosensory cortices, while the thickness of the corpus callosum was defined, at the level of the interhemispheric fissure.

### Neuropathology

#### Animal sacrifices, tissue processing and histochemical staining

Animals were sacrificed one week or three months following STZ injection by a pentobarbital overdose. Then they were immediately perfused intracardially with a 4% paraformaldehyde solution (PFA). After perfusion, whole brains were harvested and immersed in the same fixative overnight and were then plunged in a 15% and 30% sucrose solution for cryoprotection. The brain from each animal was frozen and sliced into 30 µm-thick coronal sections on a freezing Microtome. Slices were then stored at −20°C into a storage solution (Glycerol 30%, ethylene glycol 30%, and phosphate buffer 0.1 M). Then, floating sections were rinsed with 0.1 M phosphate buffer and mounted on Superfrost Plus glass slides before being stained for Glial Fibrillary Acidic Protein (GFAP, 1/30000, Dako), ionized calcium binding adaptor molecule detection (Iba-1, 1/1000, Wako), Neuronal Nuclei (NeuN, 1/10000, Chemicon), and dopamine and cyclic AMP-regulated phospho-protein (DARPP-32, 1/2000, Santa Cruz). GFAP and Iba-1 antibodies permit to detect activated astrocytes and microglial cells respectively, NeuN antibody recognizes nuclei of neurons, and DARPP-32 inform about the functional state of gabaergic neurons. The brain samples were pretreated with hydrogen peroxide (0.3%) and were bathed into a Phosphate Buffer's (0.1 M) Saline's (0.9%) and Triton's (Sigma 100×, 0.2%) solution (PBS + Tx) with Normal Goat Serum (NGS) (Sigma G6767, 4.5%). Then, during two days, the samples were incubated in a solution of PBS + Tx with NGS (3%) and the primary antibody. The samples were bathed for one hour in a solution PBS + Tx with NGS (3%) with the 1/1000 biotinylated anti-mouse and anti-rabbit secondary antibodies (VECTOR BA-9200 and BA-1000 respectively). Next, the signal was amplified by using avidine–biotine–vectastain complex standard (ELITE PK 6100 0.4%). Final reaction made use of diaminobenzidine (DAB)-nickel (VECTOR, SK-4100), DAB alone or VIP (VECTOR, SK-4600) for two minutes as chromogen (gray–black, brown or purplish pink stains, respectively) and some sections were counterstained with haematoxylin. Negative controls were performed by omitting the primary antibody in the procedure. No labeling was observed for any negative controls.

#### Quantification of neuronal density on NeuN stained sections

NeuN labeled coronal sections were viewed using an AxioPlan2 microscope equiped with an x/y/z movement-sensitive stage and video camera connected to a computer. Neurons were counted by applying systematic unbiased sampling using the Optional Fractionator probe of Mercator (ExploraNova, La Rochelle, France) in the striatum and septum. For each cerebral region, NeuN stained cells were counted on the left side of the brain in five sections at 270 µm intervals. A counting frame of 100×100 µm was employed, and the number of counted cells was extrapolated to the overall surface of each cerebral structure. The total number of cells was then calculated thanks to the Abercrombie correction factor.

#### Analysis of striatal atrophy

Striatal atrophy was evaluated by immunohistochemistry using DARPP-32 antibody. This latter informs about the functional state of gabaergic neurons and labels the striatum. Coronal DARPP-32-labelled sections were digitized using a MicroComputer Imaging Device densitometry system model (MCID®, Linton, Cambridge, UK). The striatal volume of each animal was quantified and outlined using a digitizing tablet on six slices. The most rostral slice was the slice corresponding to coordinates anteroposterior +2.3 mm [37]. The lateral and dorsal borders consisted of the corpus callosum and the ventral limit was defined by the commissura anterior. The sixth slice corresponded to coordinates anteroposterior +0.1 mm [37]. The lateral borders were the lateral ventricles and the corpus callosum, the dorsal border was the corpus callosum and the ventral limit was defined by a line from above the commissura anterior. Volumes were then calculated by taking into account the number of series and the thickness of slices using the Cavalieri method [38].

### Statistical analysis

Statistical analyses were performed using Statistica 7 Software (Statsoft, France). The significance of between-group differences was tested by Mann-Whitney's test or by analysis of variance (ANOVA) with repeated measures followed by Tukey post hoc tests. Statistical significance was set to the p≤0.05. Results were expressed in figures as mean ± standard error of the means.

## Results

### STZ induces septal and white matter atrophy

Rats that received a bilateral icv administration of 1 and 3 mg/kg of streptozotocin were followed-up for three months by MRI. Visual inspection of the images revealed alterations such as ventricular dilation, septal and white matter atrophy three months following the injection of either 1 or 3 mg/kg dose (Fig. 1A). Visual inspection of the MR images did not reveal any other lesions in other brain regions at any of the time points studied.

We evaluated the volume and thickness of regions adjacent to the ventricles (septum, striatum, parietal cortex, and white matter). This analysis revealed a 83% atrophy of the septum in the 3 mg/kg icv-STZ rats at three months post-injection, as compared to age-matched vehicle-treated control animals (Fig. 1B, ANOVA F_time*group_(2,14) = 120.3, p<0.001). The septal atrophy also occurred in the 1 mg/kg icv-STZ rats three months after the treatment, but to a lesser extent (19% decrease as compared to controls, ANOVA F_time*group_(2,14) = 120.3, p<0.05). No septal atrophy was detected one week after STZ injection, regardless of the dose used (Fig. 1B, ANOVA F_time*group_(2,14) = 0.7, NS).

The thickness of corpus callosum was reduced by 66% in the 3 mg/kg icv-STZ rats at three months as compared to age-matched control animals (Fig. 1C, ANOVA F_time*group_(2,14) = 8.9, p<0.005). On the other hand, with the 1 mg/kg dose, no changes of corpus callosum thickness could be detected three months after icv-STZ administration (Fig. 1C, NS). Also, no changes of corpus callosum could be detected one week after STZ injection whatever the dose (Fig. 1C, NS).

The striatal volume (Fig. 1D) and cortical thickness (data not shown) were not affected by the STZ administration at any of the doses and time-points studied (NS).

### STZ affects septal neurons

**Figure 2:**
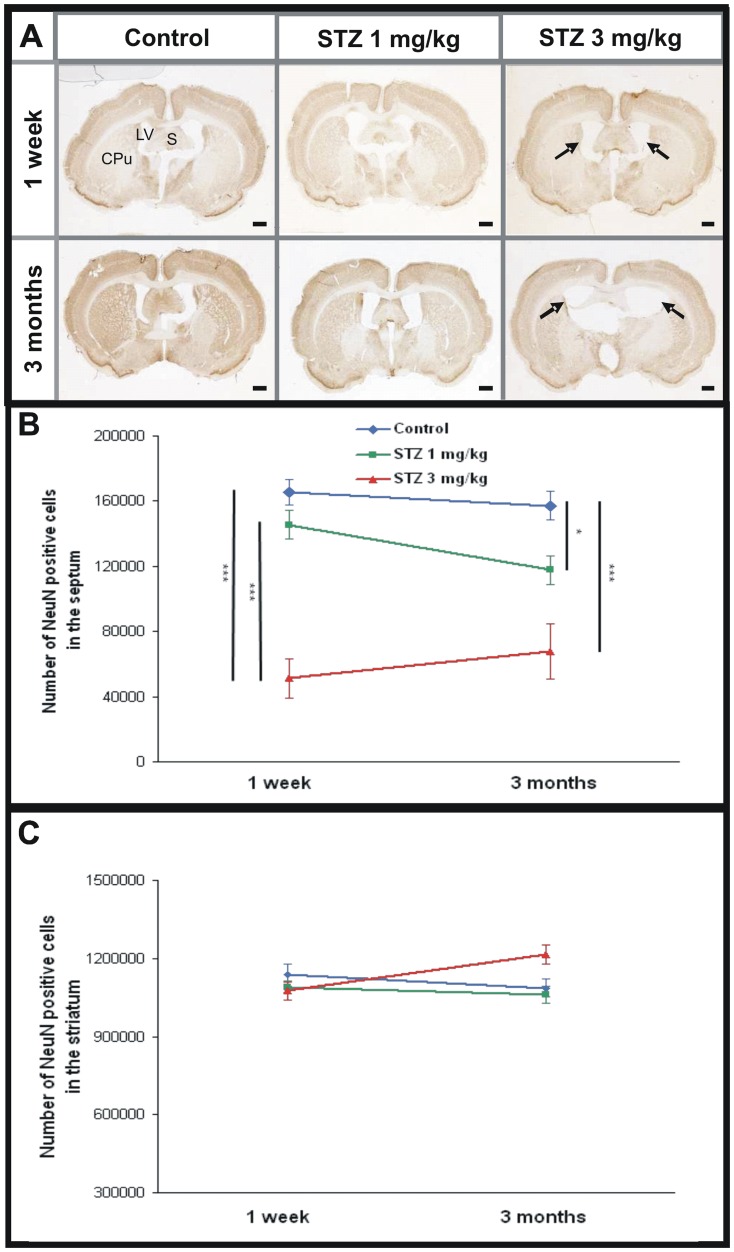
Evaluation of neuronal density on NeuN stained sections after icv- streptozotocin. A. Histological sections showing the septum (S), striatum (Cpu) and lateral ventricles (LV) in the control, 1 mg/kg and 3 mg/kg streptozotocin-treated animals, one week and three months post injection. Dilation of ventricles and septal alterations are visible in the 3 mg/kg-treated group (arrows). B. Septal neurons density started to decrease one week post injection in the 3 mg/kg group but not in the 1 mg/kg animals. This septal neuronal alteration worsened with time and was highlighted in 1 mg/kg and 3 mg/kg streptozotocin-treated animals three months following the administration of the toxic. C. No modifications of the striatal NeuN labeling density were highlighted one week or three months post injection in control group and in both streptozotocin-treated group. Values are mean±SEM. *: p<0.05; ***: p<0.005. Scale bars = 2 mm.

The integrity of septal neurons was further evaluated using NeuN immunohistochemistry. This latter highlighted a severe decrease of NeuN stained cells in the septum of STZ-treated animals one week post injection in the 3 mg/kg STZ-treated animals (decrease of 69%, Fig. 2B, ANOVA F_time*group_(2,27) = 2.0, p<0.001) but not in the 1 mg/kg group (Fig. 2B, NS). The septal neuronal alteration was severe three months post injection at both doses (decrease of 25% and 57% at 1 mg/kg and 3 mg/kg, respectively compared to control group, Fig. 2B, ANOVA F_time*group_(2,27) = 2.0, p<0.05 and p<0.001, respectively).

### Striatum is not vulnerable to icv-STZ administration

**Figure 3:**
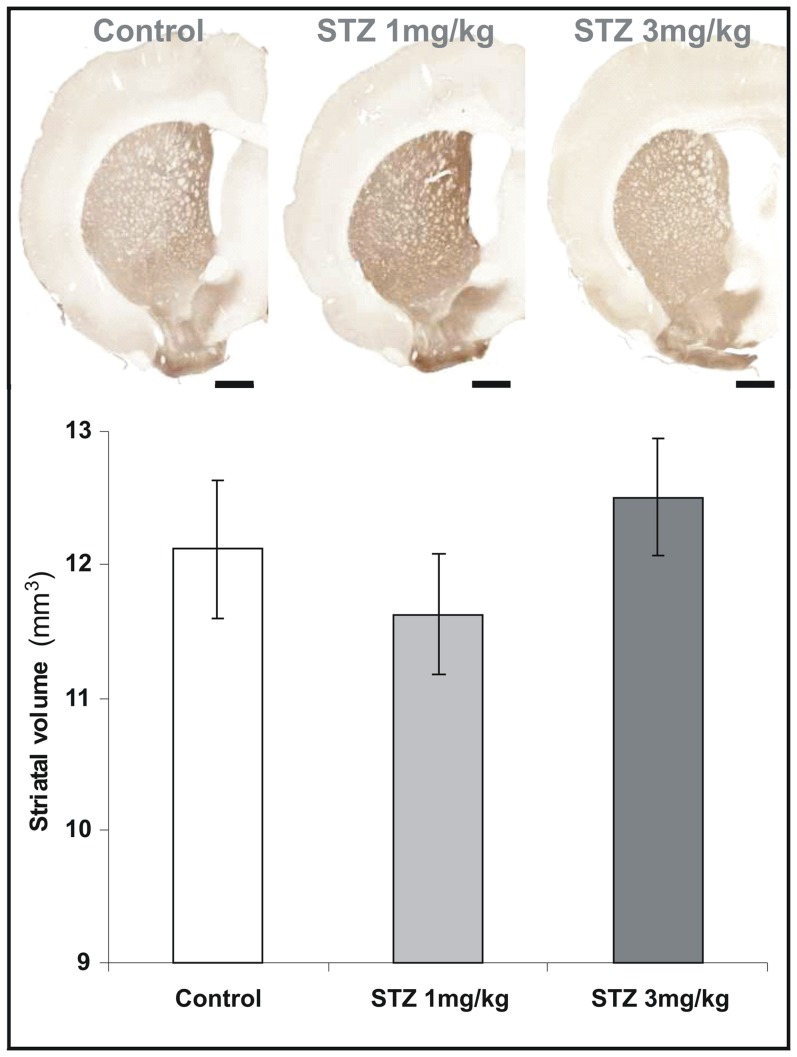
Evaluation of DARPP-32 positive regions. DARPP-32 positive striatal volumes were not modified in all groups three months following the injection (Mann-Whitney U Test, NS). Values are mean±SEM. Scale bars = 2 mm.

Neuronal density, evaluated by NeuN immunohistochemistry, was not changed in the striatum in STZ-treated animals compared to controls (Fig. 2C, ANOVA F_time*group_(2,29) = 4.9, NS) whatever the doses and time points. DARPP-32 detection also showed no alterations of striatal volumes in the 3 or 1 mg/kg treated animals compared to controls (Fig. 3; 12.5±0.4 mm^3^, 11.6±0.5 mm^3^, and 12.1±0.5 mm^3^ for the 3 mg/kg, 1 mg/kg, and control groups, respectively; Mann-Whitney U Test, NS).

### STZ induces a localized inflammation process

**Figure 4:**
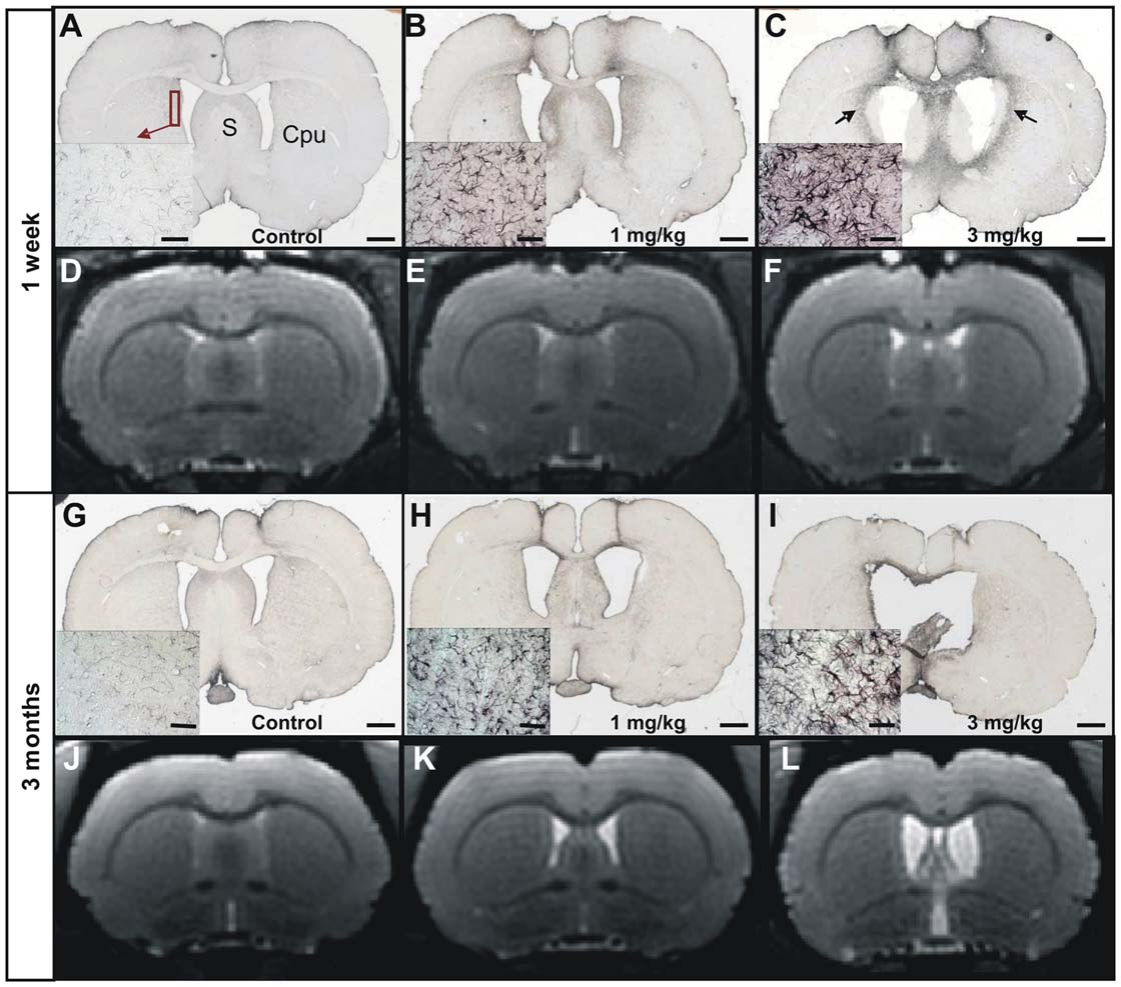
Evaluation of the astrogliosis in control (A, G), 1 mg/kg (B, H) and 3 mg/kg (C, I) icv-streptozotocin treated groups, seven days (A–C) and three months (G–I) post injection. MRI sections corresponding to the histological sections are shown in D–F (one week post- injection) and J–L (three months post-injection). Low and high (left insets) magnification images showing Glial Fibrillary Acidic Protein (GFAP) staining. A severe astrogliosis was detected in the 3 mg/kg treated group (halo of activated astrocytes in C (arrow), insets in C and I). A slight astrogliosis could be detected in the 1 mg/kg animals (B, H). The inflammation process was not obvious on MR images at both times and in all groups (D–F for one week post-injection, and J–L for three months post-injection). However, dilation of the ventricles was clearly visible on these same images in STZ-treated-animals. S: Septum. Cpu: Striatum. Scale bars for low magnification images = 2 mm and scale bars for insets = 50 µm.

**Figure 5:**
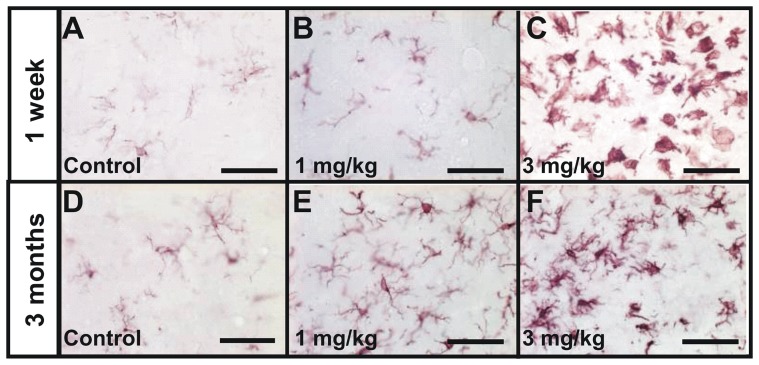
Evaluation of the microgliosis in control (A, D), 1 mg/kg (B, E) and 3 mg/kg (C, F) icv-streptozotocin treated groups, seven days (A–C) and three months (D–F) post injection. Pictures show Iba-1 staining (microgliosis). A severe microgliosis was detected in the 3 mg/kg treated group in the regions adjacent to the lateral ventricles (C and F). In the 1 mg/kg group, this microglial activation was less severe at both time points (B and E). Other brain regions did not display abnormal hyperactivated gliosis. Scale bars = 50 µm.

Inflammation processes were evaluated by GFAP (astrocytosis) and Iba-1 (microgliosis) staining. The 3 mg/kg treated animals displayed a severe inflammation process characterized by a halo of GFAP-stained activated astrocytes that involved the septo-striatal region (Fig. 4C). This halo (Fig. 4C, arrows) surrounded dorsal and ventral lateral septum and the part of the striatum directly adjacent to the lateral ventricles, filled-in with activated microglial cells (Fig. 5C). This inflammation process was already initiated one week after STZ administration (Fig. 4C). In the 1 mg/kg group, the inflammation process was less severe than in the 3 mg/kg group (Fig. 4B–C and Fig. 5B–C for the one week time point and Fig. 4H–I and Fig. 5E–F for the three month time point). It was associated to an astrocytic reaction and a slight microglial reaction close to the site of injection, i.e affecting a part of the striatum and lateral septum, that could be detected three months post-injection (Fig. 4H–I, Fig. 5E–F). The inflammation process highlighted by immunohistochemistry in STZ-treated animals was not detected by MRI (Fig. 4D–F and 4J–L). This can be explained by an insufficient T2-weighting of the recorded images as the latter were mainly recorded to evaluate cerebral atrophy.

## Discussion

Intracerebroventricular administration of streptozotocin in rodents is used to create an animal model of dementia. The rationale for this model is that STZ induces alterations of insulin/IGF pathway and glucose metabolism [10], [11], [12] as well as oxidative stress [15] leading to cerebral alterations and behavioral impairments [15], [21]. Such model is used to evaluate the effect of various drugs [13], [21], [22], [23], [24], [25], [26], [27], [28], [29], [30], [32], [33]. However, a wide range of doses are used in the literature in icv-STZ rodents without specifying the effects of doses and the location of the lesions induced. In the literature, the extension of the lesions induced by STZ is merely described, and it is often assumed that the toxic induces diffuse lesions that affect most of the brain regions. Here, we used 3D-MRI and histology to screen the whole brain of rats after bilateral intracerebroventricular administration of low (1 mg/kg) and high doses (3 mg/kg) of STZ. We present evidence that icv-STZ induces focal lesions that involve septal and corpus callosum regions and are associated with a neuronal loss and an inflammation process. A good consistency was detected between results from *in vivo* evaluation of brain volume by MRI and quantification of neuronal loss, except in the septum in the 3 mg/kg group, one week after the STZ injection. At this time point, the neuronal loss in the septum was significant while no alteration was seen by MRI at this stage. This can be explained by the lower sensitivity of MRI method as compared to neuronal counting and also by a swelling of the septal tissue because of the severe inflammation at this early stage. A similar lack of perfect relationship between measures of cerebral atrophy detected by MRI and histological evaluations is commonly reported in the literature [39], [40].

The lesions of the corpus callosum have never been described before, although white matter alterations at the level of the fornix were already reported in icv-STZ models [41]. During dementia such as Alzheimer's disease in humans, there is also a severe atrophy of the corpus callosum that is reported and this latter is correlated to cognitive impairments [42], [43]. The model can thus mimic these alterations. On the contrary, the lesions of the septum that we highlighted are consistent with a previous report showing a 40% reduction in the weight of the septum after icv-STZ administration [44], The septum has cholinergic projections on several cortical regions including the entorhinal and cingulate cortex [45] and on the hippocampus [46], [47]. These connections form the septohippocampal system which is implicated in spatial learning, short-term memory and attention [48]. The septal atrophy highlighted in our study suggests that icv-STZ rats are a model of cholinergic denervation. Such denervation, septal alterations, but also white matter alterations could be responsible for behavioral alterations reported in icv-STZ models [7], [11], [15], [20], [21], [49]. Other models of septohippocampal denervations have been reported in the past. For example the use of 192 IgG-saporin, which selectively destroys cholinergic neurons, can lead to septohippocampal lesions [50]. Icv-STZ might be less specific to cholinergic neurons but might also provide a wider range of persistent lesions including expanded and dose-dependant neuroinflammation, a process that is currently associated to neuronal loss in neurodegenerative diseases [51]. In other cholinergic depletion models, inflammation was limited to the site of injection, namely the medium septum and the diagonal band of Broca in most cases [52], [53].

We also highlighted the difference of lesions induced by low and high doses of STZ. At low dose, the corpus callosum was not atrophied whatever the time point, the neuronal loss and atrophy of the septum was not detected one week after Icv-STZ administration and was moderate at three months post administration, and inflammation was moderate. These processes were very severe at 3 mg/kg. The mechanisms induced at the two doses might thus be different with a chronic effect for the 1 mg/kg dose and an acute toxic effect leading to acute neuronal death for the high dose. The lower dose should thus be more interesting to evaluate neuroprotective drugs. Doses of 3 mg/kg and higher should be regarded as a way to model very aggressive neurotoxic lesions rather than subtle alterations due to small mechanistic alterations as would be expected during slowly evolving dementia processes or during aging. An intermediate dose, e.g. 2 mg/kg as tested by Shoham S. and collaborators [41], would perhaps be more relevant to evaluate some neuroprotective drugs.

Two mechanisms have been proposed to explain cerebral alterations induced by icv-STZ administration. First, icv-STZ is known to induce a severe oxidative stress [12], [13], [14], [15], [54], [55]. We speculate that the non-selective neurotoxicity and neuroinflammation induced by high doses of STZ close to the site of injection is consistent with an action of STZ via such mechanism. Oxidative stress would be less involved in the effects of STZ at low doses. Second, icv-STZ also induces an insulin resistance brain state [9], [10], [11], [12] leading to a widespread effect of STZ on energy metabolism. STZ enters the cells via the glucose transporter 2 (GLUT2). This transporter is present all over the brain [56]. Thus one can expect that, if it diffuses all over the brain, STZ leads to a reduced brain metabolism involving most brain regions and not only the septum or the corpus callosum. The location of the lesions close to the injection sites would thus be consistent with a lack of diffusion of STZ all over the brain. We can however not rule out that the STZ diffused all over the brain but that hypometabolism induced by STZ was not severe enough to induce a severe atrophy process.

As a conclusion, we characterized the lesions induced by icv-STZ in rodents. We showed that at high doses, icv-STZ induces severe neurodegenerative lesions in the septum and corpus callosum. The lesions are associated with an inflammation process that might be caused by oxidative stress. High dose icv-STZ models can thus be used to evaluate compounds that modulate oxidative stress and inflammation leading to neuronal loss. The lesions are less severe at low doses and low dose icv-STZ models might thus be more relevant to study mechanisms associated to slowly evolving dementia and neuroprotective treatments.
